# A Training Course for Psychologists: Learning to Assess (Alleged) Sexual Abuse Among Victims and Perpetrators Who Have Intellectual Disabilities

**DOI:** 10.1007/s11195-017-9476-x

**Published:** 2017-02-02

**Authors:** Petri J. C. M. Embregts, Marianne Heestermans, Kim J. H. M. van den Bogaard

**Affiliations:** 1 Department of Tranzo, Tilburg School of Social and Behavioral Sciences, Tilburg University, Warandelaan 2, 5037AB Tilburg, The Netherlands; 2 Dichterbij Science and Innovation, Gennep, The Netherlands

**Keywords:** Assessment, (Alleged) sexual abuse, Intellectual disabilities, Training course, Psychologist, The Netherlands

## Abstract

People with intellectual disabilities (ID) are at greater risk of being a victim of sexual abuse and may also be more predisposed to perpetrating sexual abuse. Although the prevalence of sexual abuse among people with ID is difficult to determine, it is clear that there are serious consequences for both victims and perpetrators, and professional support is needed. Psychologists play an important role in the assessment of sexual abuse in both victims and perpetrators and require specific knowledge and skills to execute the assessments. We therefore developed a training course for psychologists aimed at increasing their (applied) knowledge of sexual abuse and the related assessment process in people with ID. In a five-day training course, sessions focusing on theories about diagnostic models were combined with sessions focusing on the assessment of sexual abuse of victims and perpetrators. The effectiveness of the training course was determined in terms of (applied) knowledge via the administration of a study-specific questionnaire including a hypothetical case vignette before, immediately after, and six months after completion of the course. The results show that the knowledge of the psychologists related to sexual abuse and the assessment process for sexual abuse increased significantly, and remained above pre-test level at six-month follow-up. These results are promising, but more research is needed to see if the increased (applied) knowledge in turn leads to application in practice and better care for both victims and perpetrators.

## Introduction

People with intellectual disabilities (ID) are more at risk for sexual abuse than people without ID [1]. Sexual abuse often has severe consequences for both victims (e.g., psychological trauma) [2] and perpetrators (e.g., stigma) [3]. However, sexual abuse frequently goes undisclosed and appropriate sexual behavior and development is not always supported [4–6]. These circumstances—together with personal factors like a poor understanding of social conventions [4], and relatively fewer long-term and poorer personal relationships [7, 8]—may predispose people with ID to sexual abuse either as a victim or as a perpetrator.

Professionals play a significant role with regard to the care surrounding (alleged) sexual abuse. They have an important task in recognizing physical, behavioral and emotional signs of abuse in people with ID [9, 10] and to determine a subsequent course of action. However, the support provided by professionals is expected to be optimal only when they are sufficiently informed about the characteristics and care demands of people with ID and aware of available assessment and treatment possibilities regarding sexual abuse. Unfortunately, the majority of professionals providing support for people with ID are not well informed in this matter [11, 12].

Several researchers have developed training courses to address the gaps in knowledge and skills of professionals regarding sexual abuse in people with ID [13, 14]. However, to the best of our knowledge no studies have evaluated such training courses for psychologists, specifically aimed at the assessment of sexual abuse. Assessment of sexual abuse is important in order to: clarify the event(s) of sexual abuse and its consequences, clarify the developmental history to explain the behavior and estimate the risk of reoccurrence [15, 16], and support individuals with ID to disclose the abuse in a nonthreatening way [9]. Despite the importance of assessment of sexual abuse, research shows that most psychologists received only limited training in the area of sexuality and that these gaps in knowledge and skills are not being filled through continuing education [17].

In the present study, we therefore evaluated a five-day training program developed for psychologists to detect and assess (alleged) sexual abuse among people with ID who have been either the victim or the perpetrator of sexual abuse. The aim of the training course *Assessment of (Alleged) Sexual Abuse among Victims and Perpetrators with ID* was to increase knowledge of factors important for the assessment of sexual abuse and to increase the ability to apply this knowledge to situations derived from practice for both possible victims and perpetrators.

## Methods

### Participants

The study was approved by the scientific and ethics committee of one of the participating organisations. Fourteen professionals of four different organisations started the training course. These professionals all gave their permission to participate in the research. Written consent was received from all of them. Participants who missed more than half a day of the training course (*n* = 3) were not included in the study. Using a pre-test post-test design with a six-month follow-up, eleven participants (78.6%) completed the questionnaire at pre-test and post-test and 7 participants (50.0%) completed the questionnaire at follow-up. Analyses were based on those 7 female psychologists, in the age between 26 and 40 years, working at four different ID-services in the Netherlands.

### Procedure

The training course *Assessment of (Alleged) Sexual Abuse among Victims and Perpetrators with ID* was conducted from October 2012 till June 2013. After agreement with their manager, psychologists concluded their enrolment via completing an application form, on a voluntary basis. Participants were pre-tested at the beginning of the first day of the training course, post-tested at the end of the fourth day of the training course and tested at follow-up six months after completion of the training course.

### Training Course

The training course *Assessment of (Alleged) Sexual Abuse among Victims and Perpetrators with ID* was developed through the years by the authors in collaboration with clinical experts. During the year prior to the current study, pilot evaluations of the training course were conducted by the authors in collaboration with clinical experts involving two training groups. Subsequently, the training course was adjusted to make the central points of the course more apparent. The content of the final training course is outlined in Table 1. A range of teaching and training methods and materials were used including lecture, whole-group discussion, small-group discussion, small-group work, analysing video materials, role play and discussion of homework exercises.

**Table 1:** Outline of the training course *Assessment of (Alleged) Sexual Abuse among Victims and Perpetrators with Intellectual Disabilities*: Theoretical topics and content

Theoretical topics	Content
General diagnostic models (1 day)	Hypothesis-testing model
	SORC model^a^
Assessment of victims (2 days)	Definition of sexual abuse: victims
	Classification of types of sexual abuse
	Signs of sexual abuse
	Definition and criteria for trauma
	Assessment instruments and their use
Assessment of perpetrators (2 days)	Definition of sexual abuse: perpetrators
	Explanations for sexual abuse
	Assessment instruments and their use
Case study (1 day at follow-up)	Application of the theoretical content to a case study put forward by the participants

^a^SORC model = Stimulus-Organism-Response-Consequences model [18]

### Instrument

#### Sexual Abuse Knowledge Questionnaire-Victims and Perpetrators

The *Sexual Abuse Knowledge Questionnaire*-*Victims and Perpetrators* (SAKQ-VP) was developed by the authors in collaboration with clinical experts for purposes of the present study. The content of this questionnaire was based on the aims of the training course *Assessment of (Alleged) Sexual Abuse among Victims and Perpetrators with ID*. The questionnaire, divided in Part A and B, was developed in line with the Taxonomy for learning, teaching, and assessing by Anderson, Krathwohl, and Bloom [19] which holds that applying knowledge is a more complex category of cognitive processing than merely remembering and understanding (original taxonomy from Bloom and colleagues [20]). Participants could score a total of 15 points for part A and B.

Part A of the questionnaire addresses the recalling and understanding of the assessment of sexual abuse. The main topics addressed in Part A of the questionnaire were: (1) knowledge of general diagnostic models (e.g., *Explain what the hypothesis*-*testing model is used for?*); (2) knowledge of victim assessment (e.g., *What are the differences in trauma theories for people with and without ID?*); (3) knowledge of perpetrator assessment (e.g., *Which environmental factors reinforce and maintain sexually offensive behavior of people with ID?*). Part A consists of 11 questions: 10 multiple-choice questions and 1 open-ended question. Respondents could score a maximum of 11 points.

Part B focusses on applying the knowledge of the assessment of sexual abuse. A hypothetical case vignette is presented to the participants. To gain insight into the participant’s interpretation of the problem situation and their planned manner of acting, the participant is asked to answer four multiple-choice questions (e.g., *Which of the steps below would you take to analyse the problem and in which order?*). Respondents could score a maximum of 4 points.

### Statistical Analysis

To compare the mean scores of the participants on the SAKQ-VP between pre-test, post-test and 6-month follow-up, a non-parametric Friedman’s test and post hoc Wilcoxon signed-rank tests were used.

## Results

The results on the SAKQ-VP are shown in Table 2. Friedman’s test revealed that there were statistically significant differences between the total scores at the three measuring points with respect to the questions on the SAKQ-VP [χ^2^ (2) = 9.56, *p* = .008]. Post-hoc analysis using Wilcoxon signed-rank tests revealed a significant difference between pre-test and post-test (*Z* = −2.37, *p* = .018) and pre-test and follow-up (*Z* = −2.21, *p* = .027). The scores at follow-up did not differ significantly from the scores at post-test (*Z* = −.51, *p* = .611) and thus remained stable.

**Table 2:** Pre-test, post-test and follow-up scores on the SAKQ-VP for Part A, Part B and the total test

		Recalling and understanding (part A)			Applying (part B)			Total (part A & B)		
	*N*	*M*	*SD*	Min–max	*M*	*SD*	Min–max	*M*	*SD*	Min–max
Pre-test	7	3.07	1.26	1–5	0.86	0.69	0–2	3.93	1.16	3–6
Post-test	7	5.07	1.15	4–7	1.57	0.98	0–3	6.64	1.27	5–9
Follow-up	7	5.14	1.56	4–8	1.29	0.95	0–3	6.43	2.16	5–11

## Discussion

Although individuals with ID are more at risk for sexual abuse than individuals without ID [1], few training courses and methodologies are available for psychologist to support people with ID as victims or perpetrators of sexual abuse. The current study forms an important first step in addressing this gap, to our knowledge it is the first to evaluate a training specifically aimed at assessment of sexual abuse by psychologists. As a result of the offered training, findings of the current study show that psychologists’ (applied) knowledge regarding assessment of sexual abuse for both victims and perpetrators with ID increases significantly after they have participated in the training course. Moreover, these effects persist until 6 months after the training.

Previous studies on training programs for support staff were evaluated in terms of knowledge of and attitudes about sexual abuse [13, 14]. However, in line with the notion that the application of knowledge is a more complex cognitive process rather than merely recalling and understanding [19], applied knowledge was also assessed in the current study. While the study was strengthened by the inclusion of an applied knowledge measure, other quality indicators are needed to determine if the changes in (applied) knowledge lead to significant behavior changes of the psychologists in everyday practise. In future research, extended follow-up interviews could be conducted with psychologists and their co-workers or supervisors to determine how the training course has influenced their practices with regard to (alleged) sexual abuse.

Furthermore, although we found significant increases in (applied) knowledge following the training, the number of respondents was small and the scores were not at their maximum, suggesting that psychologists may require, next to a classroom training, even more intensive coaching and supervision. As shown in a meta-analysis of training programs for support staff working with people with ID, a combination of in-service training (classroom/workshop) and coaching on the job may be the most effective [21]. Such a training format would be similarly beneficial for psychologists, and future studies could assess the combination of classroom/workshop lessons with coaching on the job in training the assessment of sexual abuse.

Despite limitations of the current study regarding the transfer of knowledge, it seems likely that enhanced knowledge of sexual abuse and the assessment process will lead to greater sensitivity to potential cases of sexual abuse in practice and to a more efficient assessment process following a disclosure. Assessment is an important step in the treatment of the consequences of sexual abuse as well as the prevention of sexual abuse by potential perpetrators. Trained psychologists will be better equipped to confirm/disconfirm their suspicions of sexual abuse and to deal with cases of (alleged) sexual abuse, which will ultimately benefit wellbeing of people with ID.
